# Deeply digging the interaction effect in multiple linear regressions using a fractional-power interaction term

**DOI:** 10.1016/j.mex.2020.101067

**Published:** 2020-09-16

**Authors:** Xinhai Li, Baidu Li, Guiming Wang, Xiangjiang Zhan, Marcel Holyoak

**Affiliations:** a Key Laboratory of Animal Ecology and Conservation Biology, Institute of Zoology, Chinese Academy of Sciences, Beichen West Road, Beijing 100101, China; b University of Chinese Academy of Sciences, Yuquan Road, Beijing 100049, China; c York University, 4700 Keele Street, Toronto, Ontario M3J 1P3, Canada; d Department of Wildlife, Fisheries and Aquaculture, Mississippi State University, Mississippi State, MS 39762-9690, USA; e CAS Center for Excellence in Animal Evolution and Genetics, Chinese Academy of Sciences, Kunming 650223, China; f Department of Environmental Science and Policy, University of California, 1 Shields Ave., Davis, CA 95616, USA

**Keywords:** Fractional-power interaction regression (FPIR), Multiple linear regression, Nonlinearity, InteractionFPIR, R package

## Abstract

In multiple regression Y ~ β_0_ + β_1_X_1_ + β_2_X_2_ + β_3_X_1_ X_2_ + ɛ., the interaction term is quantified as the product of X_1_ and X_2_. We developed fractional-power interaction regression (FPIR), using βX_1_^M^ X_2_^N^ as the interaction term. The rationale of FPIR is that the slopes of Y-X_1_ regression along the X_2_ gradient are modeled using the nonlinear function (Slope = β_1_ + β_3_MX_1_^M-1^ X_2_^N^), instead of the linear function (Slope = β_1_ + β_3_X_2_) that regular regressions normally implement. The ranges of *M* and *N* are from -56 to 56 with 550 candidate values, respectively. We applied FPIR using a well-studied dataset, nest sites of the crested ibis (*Nipponia nippon*).We further tested FPIR by other 4692 regression models. FPIRs have lower AIC values (-302 ± 5003.5) than regular regressions (-168.4 ± 4561.6), and the effect size of AIC values between FPIR and regular regression is 0.07 (95% CI: 0.04–0.10). We also compared FPIR with complex models such as polynomial regression, generalized additive model, and random forest. FPIR is flexible and interpretable, using a minimum number of degrees of freedom to maximize variance explained. We have provided a new R package, interactionFPIR, to estimate the values of *M* and *N*, and suggest using FPIR whenever the interaction term is likely to be significant.

• Introduced fractional-power interaction regression (FPIR) as Y ~ β_0_ + β_1_X_1_ + β_2_X_2_ + β_3_X_1_^M^ X_2_^N^ + ɛ to replace the current regression model Y ~ β_0_ + β_1_X_1_ + β_2_X_2_ + β_3_X_1_ X_2_ + ɛ;

• Clarified the rationale of FPIR, and compared it with regular regression model, polynomial regression, generalized additive model, and random forest using regression models for 4692 species;

• Provided an R package, interactionFPIR, to calculate the values of *M* and *N*, and other model parameters.

## Graphical abstract

**Figure:**
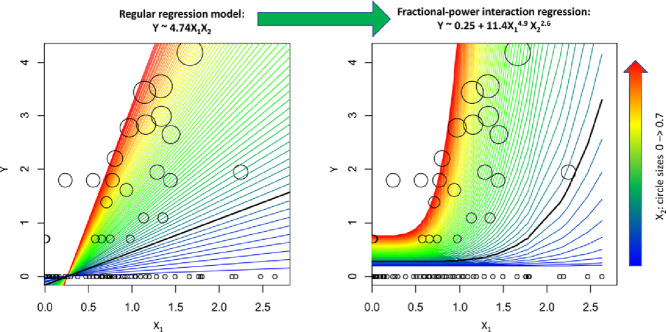

Specifications table

**Table:** 

Subject Area	Agricultural and Biological Sciences
More specific subject area	*Statistics*
Method name	*Fractional-power interaction regression (FPIR)*
Name and reference of original method	Polynomial regressions can calculate high order interaction effects such as X_1_^M^X_2_^N^, yet M and N are limited within a few integers. Compared with polynomial regressions, fractional polynomial regressions (FPRs) were proposed to model the effects of explanatory variables beyond integer exponents [1, 2]. Royston and Sauerbrei [3] further invented multivariable fractional polynomials interaction (MFPI), which can handle interactions of continuous predictors in the form of fractional polynomials. The algorithm of MFPI is available in Stata [4], which however gives limited options (i.e. −2, − 1, − 0.5, 0, 0.5, 1, 2, 3) for the powers of a predictor. The package for R, mfp [5], was designed to run MFPI, yet the function for treating interaction terms is still absent [6]. **References** [1] P. Royston, D.G. Altman, Regression using fractional polynomials of continuous covariates: Parsimonious parametric modelling, Applied Statistics 43(3) (1994) 429. [2] P. Royston, D.G. Altman, Approximating statistical functions by using fractional polynomial regression, Statistician 46(3) (1997) 411-422. [3] P. Royston, W. Sauerbrei, A new measure of prognostic separation in survival data, Stat. Med. 23(5) (2004) 723-48. [4] P. Royston, MFPIGEN: Stata module for modelling and displaying interactions between continuous predictors, Statistical Software Components S457439, Boston College Department of Economics, revised 31 Oct 2012, 2012. [5] R Core Team, R: A language and environment for statistical computing, R Foundation for Statistical Computing, Vienna, Austria, 2019. [6] Original by Gareth Ambler and modified by Axel Benner, mfp: Multivariable Fractional Polynomials, R package version 1.5.2. https://CRAN.R-project.org/package=mfp2015.
Resource availability	The R package interactionFPIR can be installed from GitHub using the code: install_github("Xinhai-Li/interaction").

## Method details

In general linear models (GLMs), the variance of the dependent variable can be explained by a number of explanatory variables, in the form of linear terms, quadratic or other high order terms, and interaction terms [1], [2], [3]. When an interaction term has a significant contribution to the model, it means the effect of one explanatory variable on the dependent variable changes depending on that of another explanatory variable. In other words, the interaction effect indicates the simultaneous influence of two variables on the dependent variable is not additive, and a nonlinear relationship is expected [4,5].

In most algorithms developed for regressions, the interaction effect is quantified as the product of two associated explanatory variables, in the form of βX_1_X_2_, where β is the coefficient, X_1_ and X_2_ are explanatory variables [4,[6], [7], [8]. In multivariable fractional polynomials interaction (MFPI), the interaction term is quantified as βX_1_^M^ X_2_^N^, but the potential values for M and N are too limited, only having eight numbers [9], which has no advantage over ordinary polynomial regressions.

To address this issue, we developed a method named fractional-power interaction regression (FPIR), using a grid search to estimate the values of *M* and *N* (each with 550 candidate values from -56 to 56) in the model Y ~ β_0_ + β_1X1_ + β_2X2_ + β_3_X_1_^M^ X_2^N^_ + ɛ. FPIR dramatically extends the shapes of interaction effect in multiple regressions.

### Data of the crested ibis for developing FPIR

FPIR can be applied for any data with several continuous variables. However, the interaction effect is complicated, so we selected ecological meaningful data to test FPIR and interpreted the results. In fact, the idea of FPIR was triggered by strong interaction effect in the model of the nest site selection by the crested ibis (*Nipponia nippon*).

The crested ibis was once critically endangered, with only two pairs left in the wild [10], and now the population has increased rapidly to over 2000 [11] and was reintroduced to many other places [12]. The majority of the wild crested ibis population is concentrated in Yang County, Hanzhong Prefecture, Shaanxi Province in Central China [13], within 95 watersheds (Supplementary Fig. 1). The average area of watersheds is 154 km^2^ [14]. Previous studies indicated that two types of wetlands were important for the birds, rice paddies and waterbodies (e.g. lakes, ponds, and rivers) [15,16], and the interaction term of two types of wetlands has a significant contribution to the habitat quality, meaning the most suitable watersheds should have certain areas of both rice paddies and waterbodies (Supplementary Fig. 1).

In FPIR, the dependent variable Y is the number of nests within each of the 95 adjacent watersheds, and it ranges from 0 to 65. One independent variable X_1_ is the area of rice paddies, vary from 0 to 12.95 km^2^. Another independent variable is waterbody area, which varied from 0 to 1.03 km^2^. Since the distributions of the three variables were concentrated at small values, we performed a log transformation (e.g. Y_t_ = log(Y + 1)) for all dependent and independent variables to make their distributions more spread out.

### Data of GBIF species occurrences for testing FPIR

We conducted massive model comparisons using the occurrence data of 4692 species (Table 1) downloaded from the Global Biodiversity Information Facility (GBIF) website, and developed multiple regressions to answer the question: how much an animal was tolerant of human impacts on our human-dominated planet, and how elevation and precipitation influenced such tolerance. We chose taxa from insects to mammals that occurred in terrestrial ecosystems. These animals do not move much, so that the occurrences could be assumed independent and identical, not like migratory birds having breeding, migratory, and overwintering occurrences. For example, Galliformes are sedentary birds with limited movement ability and Cicadidea is a family of insects that spend most of their life underground. The original occurrences were filtered as follows: potentially redundant records within 1 ha were removed, and species with < 20 occurrence records were excluded (see Supplementary Excel table).

**Table 1:** The occurrence data of taxa downloaded from GBIF website.

Class	Order	Family	Species	Occurrences	GBIF DOI
Clitellata	/	/	334	175435	https://doi.org/10.15468/dl.4vlmaw
Insecta	Hymenoptera	Formicidae	2153	290125	https://doi.org/10.15468/dl.c9o5mh
Insecta	Hemiptera	Cicadidae	174	14585	https://doi.org/10.15468/dl.mqaniq
Arachnida	Araneae	Salticidae	281	48792	https://doi.org/10.15468/dl.383zw0
Amphibia	Anura	Hylidae	348	193922	https://doi.org/10.15468/dl.qjwkh1
Reptilia	Squamata	Colubridae	295	128290	https://doi.org/10.15468/dl.okmmxx
Reptilia	Squamata	Scincidae	595	244326	https://doi.org/10.15468/dl.nnyj0o
Aves	Galliformes	/	256	1151250	https://doi.org/10.15468/dl.lwji3z
Mammalia	Lagomorpha	/	50	198132	https://doi.org/10.15468/dl.oqcwcl
Mammalia	Artiodactyla	/	206	283468	https://doi.org/10.15468/dl.mj88eh

We assumed that species occurrences represented their habitat preference. Environmental variables such as human footprint index [17], elevation [18], and annual total precipitation [19] were extracted on these occurrence sites.

If the species are randomly distributed on the earth, their occurrences just represent the background environmental variation. As such, we selected 15,677 evenly distributed sites on the planet's terrestrial ecosystem as a specific case showing the default species-environment relationship.

### Building FPIR

For any given dataset with a continuous dependent variable Y and two continuous explanatory variables X_1_ and X_2_, when the interaction term results in a lower AIC value, we build FPRI as:

(1) $$Y∼\beta_{0}+\beta_{1}X_{1}+\beta_{2}X_{2}+\beta_{3}X_{1}^{M}X_{2}^{N}+\varepsilon$$

To estimate the parameters *M* and *N*, we first gave them gradient values from -52.5 to 52.5, each with 55 values. The values near zero were sampled with higher density (Supplementary Fig. 2). The selection of such ranges and number of values were arbitrary, and they covered a very wide range of potential *M* and *N* values, in contrast with the default value one in regular regressions. Now we obtained 3025 combinations of the exponents of X_1_ and X_2_ in the interaction term. We fitted these 3025 corresponding models and selected the best model, defined as that with the highest R^2^ value. We further tuned the values of *M* and *N* respectively, using 10 evenly distributed values around those in the initial best model, and therefore built 100 models for comparison to obtain final values of *M* and *N*. The total potential values for *M* and *N* are 550, ranging from -56 to 56, respectively, representing a FPIR with 302,500 candidate combinations of *M* and *N*.

We built an R package interactionFPIR to estimate the values of *M* and *N*, and obtained the regression coefficients for all terms. The R^2^ values, the proportion of variance explained by the interaction term, all regression coefficients and associated p values in both FPIR and regular regressions were also recorded. The package has three functions: FPIR1twoway() estimates initial values of *M* and *N*, FPIR1twowaytune() estimates tuned values *M* and *N*, and FPIR1threeway() estimates parameters for a three-way interaction. The package can be installed from GitHub server using the code install_github("Xinhai-Li/interaction") (R package devtools is needed here).

### FPIR application for nest site selection of the crested ibis

For the nest site selection, two explanatory variables X_1_ (log-transformed areas of rice paddies) and X_2_ (log-transformed areas of waterbodies) had strong interaction effect on Y (log-transformed number of nests within watersheds), as the interaction term explained more variance than any of the main effect (linear) terms in both FPIR and the regular regression (Table 2). FPIR indicated the model Y = 0.25 + 11.4X_1_^4.9^ X_2_^2.6^ had the best performance. Compared to the regular multiple regression, the R^2^ in FPIR increased from 0.399 to 0.434; AIC decreased from 220.2 to 214.4 (Table 2).

**Table 2:** Comparison of each term between the regular multiple regression and fractional-power interaction regression (FPIR) using the nest site selection data of the crested ibis.

	Terms	Coefficients ± SE	Sum of square	*R^2^*	AIC	DF*
Regular regression model	Rice paddy	−0.04 ± 0.15	7.64	0.39	220.2	91
	Waterbody	−1.73 ± 0.98	13.53			
	Interaction	6.38 ± 1.22	15.33			
	Residual	/	50.84			
Polynomial regression†	Model	/	49.22	0.48	214.9	80
	Residual		38.13			
FPIR	Rice paddy	0.11 ± 0.13	7.64	0.43	210.4	89
	Waterbody	0.9 ± 0.59	13.53			
	Interaction	9.83 ± 1.67	18.34			
	Residual	/	47.83			

⁎ Degree of freedom of the residuals

† Fourth order polynomial regression

**Fig. 1:**
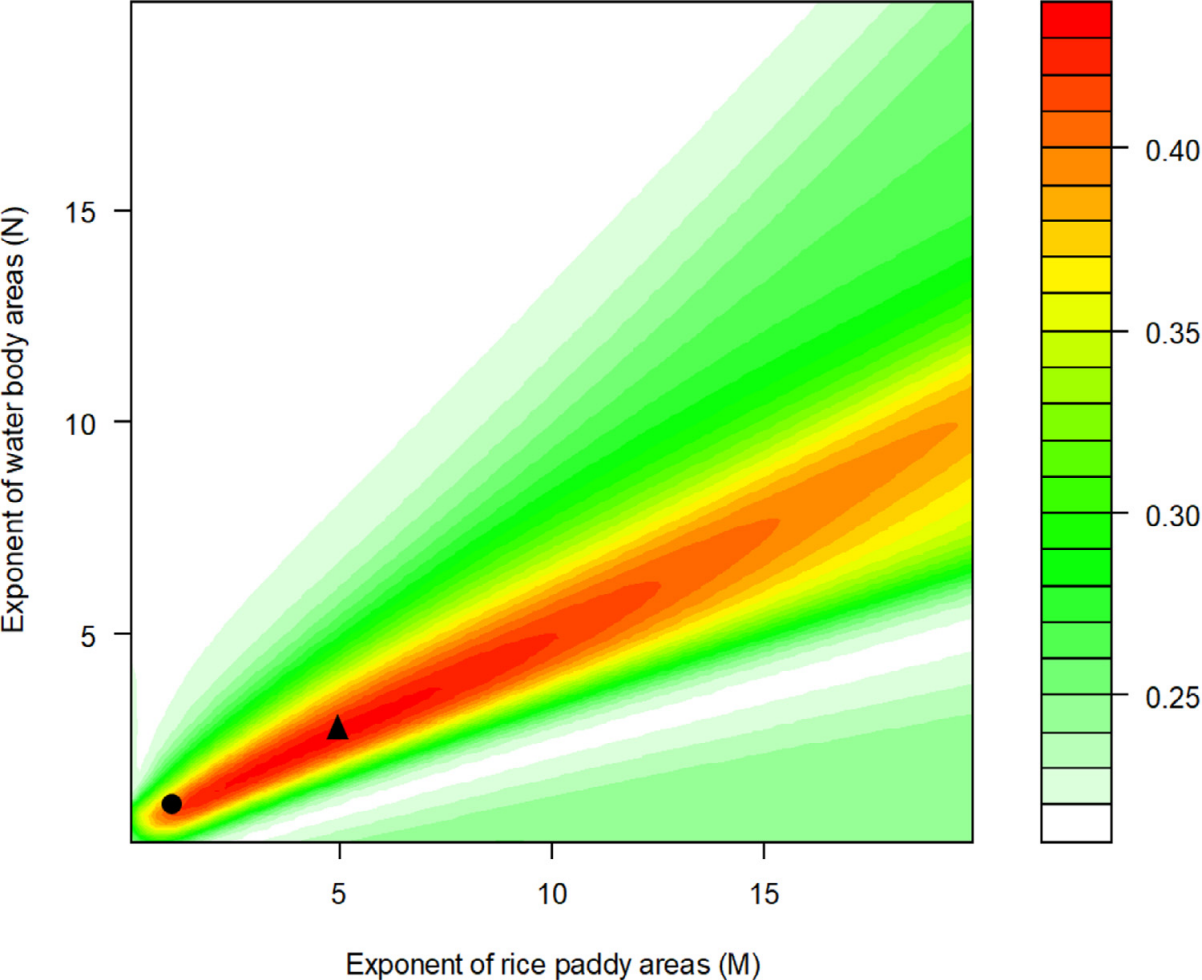
The R^2^ values (changing from green to red) of the regression model Y ~ β_0_ + β_1X1_ + β_2X2_ + β_3_X_1_^M^ X_2^N^_ + ε (*M, N* ∈ 0.1, 0.2, …, 19.9, 20) for the nest site selection of crested ibis calculated by fractional-power interaction regression (FPIR), where Y, X_1_, and X_2_ are the log-transformed number of nests, areas of rice paddies, and areas of waterbodies within each watersheds, respectively. The black dot indicates the R^2^ value of the regular regression *(M* = *N* = 1) and the black triangle indicates the R^2^ value of FPIR with the optimal *M* and *N* values.

To illustrate the model performance of all combinations of the exponents of rice paddy and waterbody, we plotted the R^2^ values at the gradients of *M* and *N* in Y ~ β_0_ + β_1X1_ + β_2X2_ + β_3_X_1_^M^ X_2_^N^ (M, N ∈ 0.1, 0.2, …, 19.9, 20). It is shown that the R^2^ value of the model performance was high when the exponents of the areas of rice paddies was about twice that of the exponents of the areas of waterbodies (e.g., 4.9 vs. 2.6) (Fig. 1). The actual interaction effect was nonlinear (Slope for Y-X_1_relationship = β_1_ + β_3_MX_1_^M-1^ X_2_^N^) at the gradient of X_2_, instead of the linear functions (Slope = β_1_ + β_3_X_2_) that the regular regression normally implements for interaction effect (see graphical abstract).

### Testing FPIR using GBIF species occurrences data

To further test FPIR, we used following regression models to study the association of human activities with elevation and precipitation at the wildlife occurrences:

(2 for FPIR) $$\text{HFI}∼\beta_{0}+\beta_{1}E+\beta_{2}P+\beta_{3}E^{M}\times P^{N}+\varepsilon$$

where *HFI* is human footprint index at the species occurrences, representing human impacts. *E* is elevation (m), *P* is annual total precipitation (mm/year), and *βs* are coefficients. To make the regression coefficients comparable, we standardized the three variables to the scale of 0–1. The model was applied to 4692 species, which are available from GBIF database (Table 1). For the purpose of comparisons, the regular regression model and fourth order polynomial regression were also applied using the same dataset. Considering the nonlinear effect of elevation and precipitation, we added the quadratic terms of *E* and *P* to FPIR, and named this model as FPIR plus (FPIRP):

(3 for FPIRP) $$\text{HFI}∼\beta_{0}+\beta_{1}E+\beta_{2}P+\beta_{3}E^{M}\times P^{N}+\beta_{4}E^{2}+\beta_{5}P^{2}+\varepsilon$$

Furthermore, we implemented two complex models for comparison: generalized additive model and random forest. *R* packages *mgcv* and *randomForest* were used for the two models. For all the 28,152 (4692 × 6) models, we recorded the adjusted *R* square values, as the index of model performance, and the Akaike information criterion (AIC) for linear models (see Supplementary Excel table).

For different species, the influence of elevation and precipitation on tolerance to human impact varied. Using regular regression models (HFI ~ E + P + E × P + ɛ), we found, in 490 species, individuals at higher elevation were significantly closer to human populations (positive coefficient of *E, p*value < 0.05). In 908 species, individuals at lower elevation were significantly closer to human populations. For the environment variable precipitation, 789 species had individuals in wetter areas that were significantly closer to human populations; compared with 679 species that had individuals in drier areas were significantly closer to human populations. The AIC values of FPIRs (-302 ± 5003.5) were lower than those of regular regressions (-168.4 ± 4561.6). The effect size of AIC values between FPIRs and regular regressions was 0.07 (95% CI: 0.04–0.10), calculated using the function effectsize() in R package effectsize [20]. The interaction term in FPIRs explained more variance than in regular regressions (Supplementary Fig. 3).

**Fig. 2:**
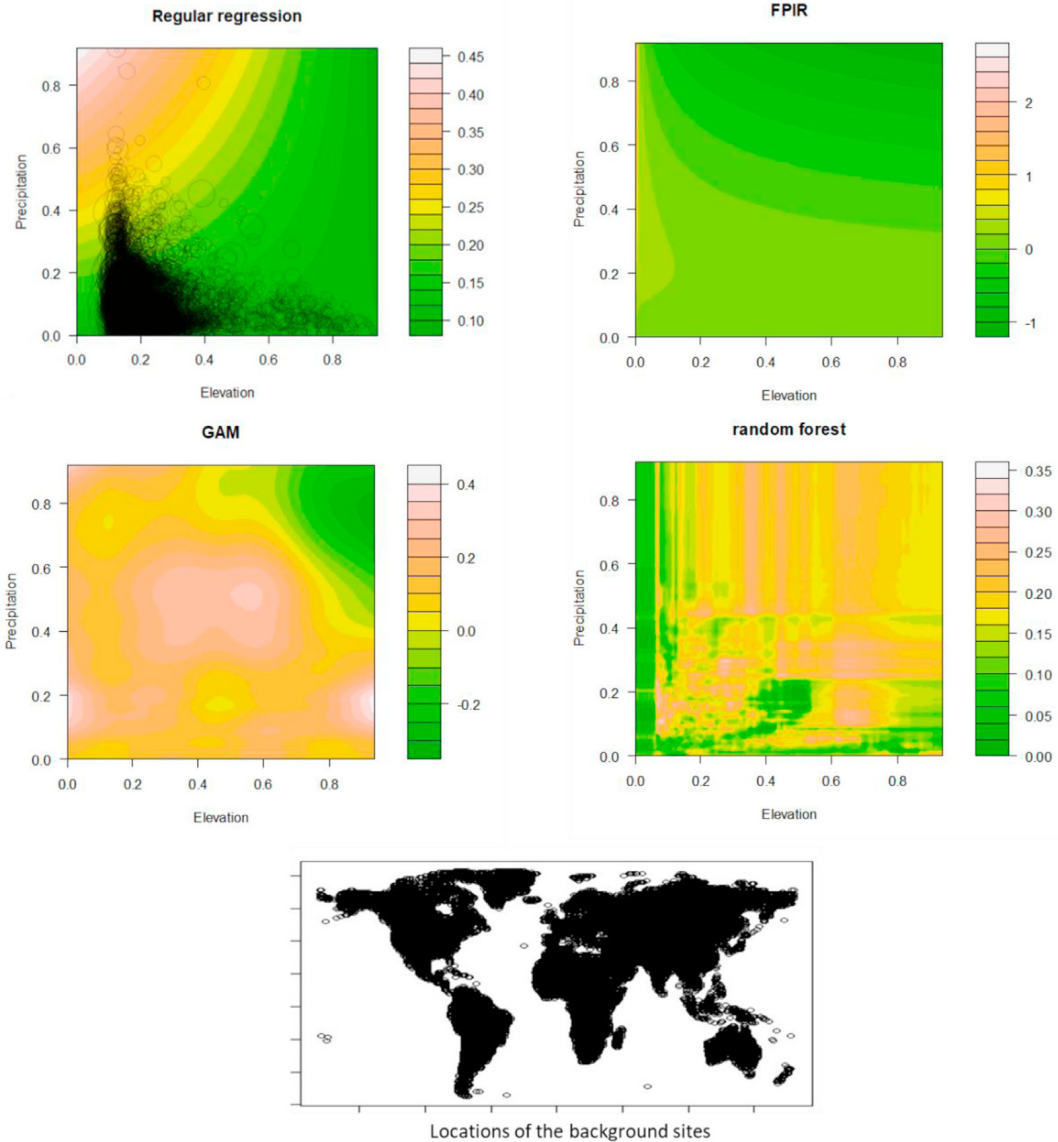
The actual human footprint index (HFI) (represented by the sizes of the circle), the predicted HFI (represented by the colors) by regular regression (HFI ~ E + P + E × P + ɛ), FPIR (HFI ~ β_0_ + β_1E_ + β_2P_ + β_3E^M^_ × P^N^ + ɛ), generalized additive model, and random forest. The standardized values of elevation (*E*) of the 15677 evenly distributed sites at the terrestrial areas on the earth is the x axis, and annual total precipitation (*P*) is the y axis. The panel at the bottom shows the locations of the 15677 sites.

If the species randomly select their habitat, the human footprint index at their occurrence sites would be independent of elevation and precipitation. We showed the pattern using 15677 evenly-distributed sites across our planet's terrestrial ecosystems (Fig. 2). In the multiple regression, elevation (*E*) explained 0.296% of the variance of *HFI*, precipitation (*P*) explained 4.27%, and the interaction term only explained 0.063% of the variance. The Pearson correlation coefficient for elevation and precipitation is -0.054. Regular regression, FPIR, GAM, and random forest quantified the relationship between human footprint index, elevation and precipitation in different patterns (Fig. 2).

**Fig. 3:**
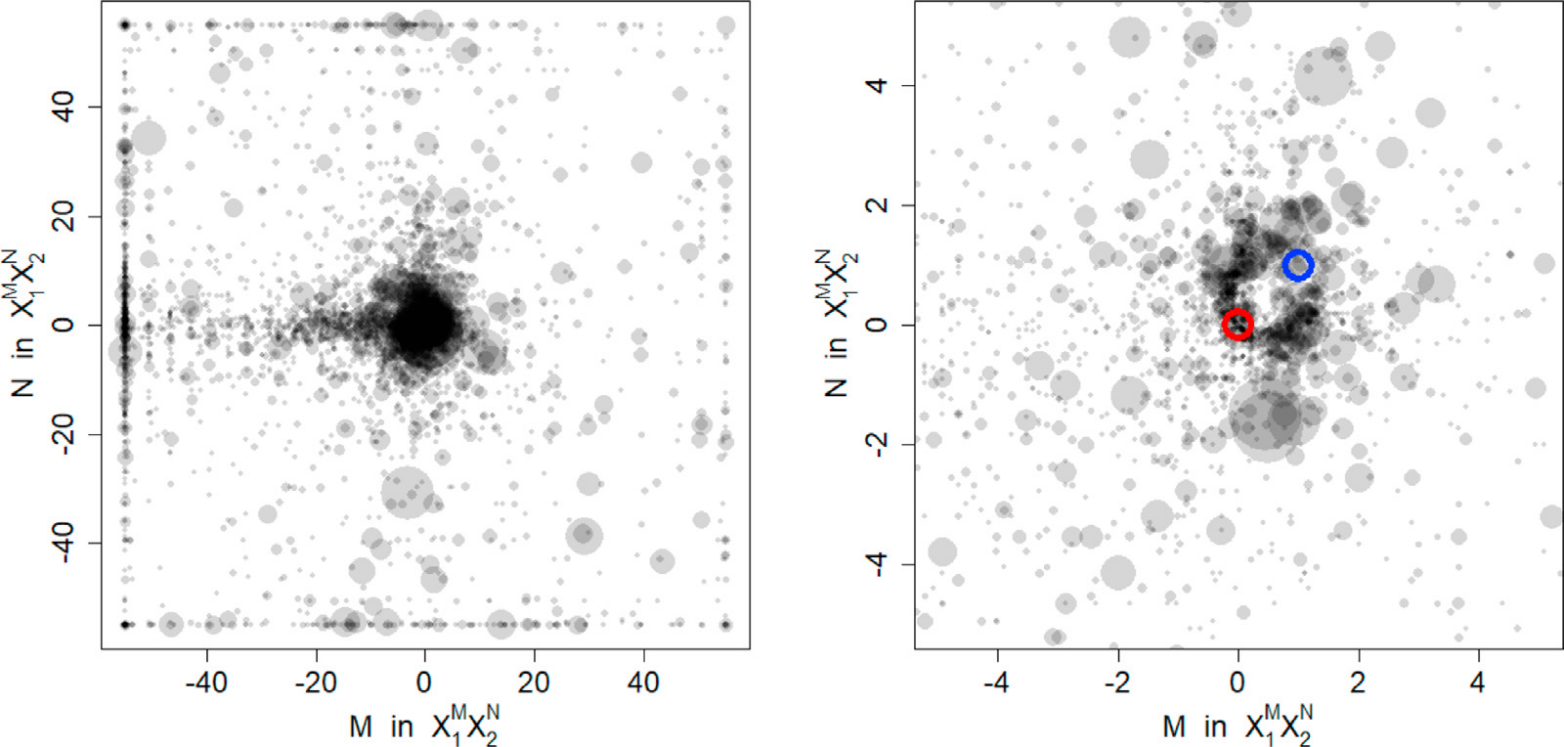
The values of parameters *M* and *N* in FPIR (HFI ~ E + P + E^M^ × P^N^ + ɛ) for the 4692 species within the range (-56 to 56, left panel) and the range (-5 to 5, right panel). The sizes of circles indicate the proportion of variance explained by the interaction term. At the right panel, the red circle shows the value zero for M and N (no interaction effect), and blue circle shows value one for *M* and *N* (traditional interaction effect) in regular regressions.

The estimated values of *M* and *N* in 4692 FPIRs (see Supplementary Excel table) were concentrated within the range from -10 to 10, yet stayed away from the default value of one (Fig. 3).

### FPIR performance compared with other models

**Fig. 4:**
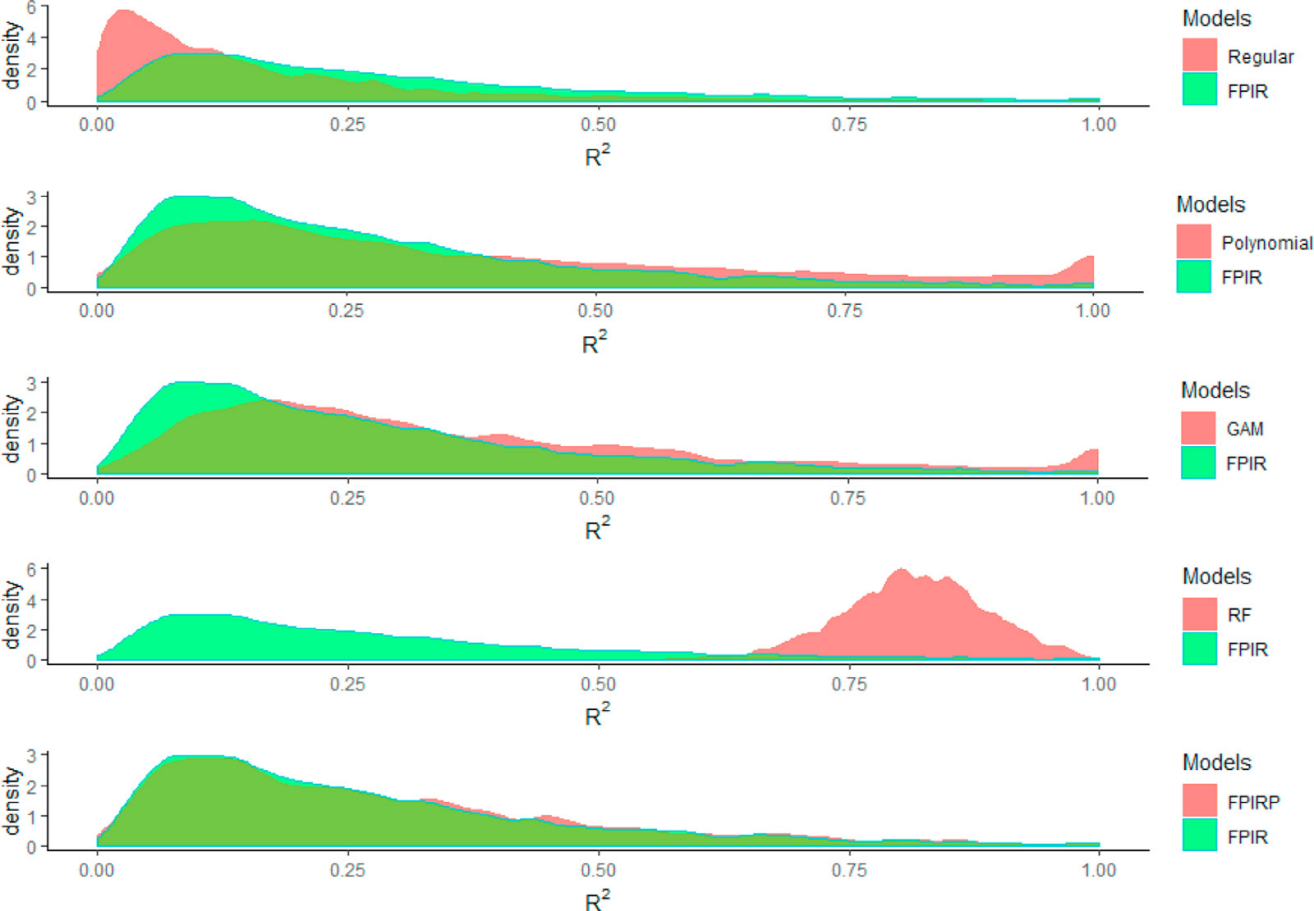
Distributions of R^2^ values for regular regression (Regular), fourth order polynomial regression (Polynomial), generalized additive model (GAM), random forest (RF), and FPIRP (FPIR plus with quadratic terms), compared with FPIR.

The average R^2^ values of FPIRs were always higher than those of regular regressions (Fig. 4). As linear models, FPIRs had lower R^2^ values than those of complex models such as polynomial regressions, generalized additive models, and random forest (Table 3). Fig. 4 further showed the distribution of R^2^ values in those models. FPIRP, which has quadratic terms, is only slightly better than FPIR (Fig. 4).

**Table 3:** The mean R^2^ values and associated standard deviations of the model HFI = f(E, P) using multiple regression, FPIR, FPIRP, fourth order polynomial regression, the generalized additive model (GAM), and random forest respectively for each of the 4692 species, and the 15,677 evenly distributed sites on earth as a background.

	Multiple regression	FPIR	FPIRP	Polynomial regression	GAM	Random forest
Focal species (N = 4692)	0.126 ± 0.164	0.267 ± 0.204	0.273 ± 0.208	0.353 ± 0.264	0.340 ± 0.243	0.813 ± 0.073
Focal species* (N = 1153)	0.217 ± 0.193	0.314 ± 0.233	0.311 ± 0.233	0.399 ± 0.285	0.367 ± 0.246	0.840 ± 0.069
Background	0.046	0.109	0.109	0.136	0.170	0.745

⁎ The species with significant (α = 0.05) interaction effect (based on multiple regression) were selected.

**Fig. 5:**
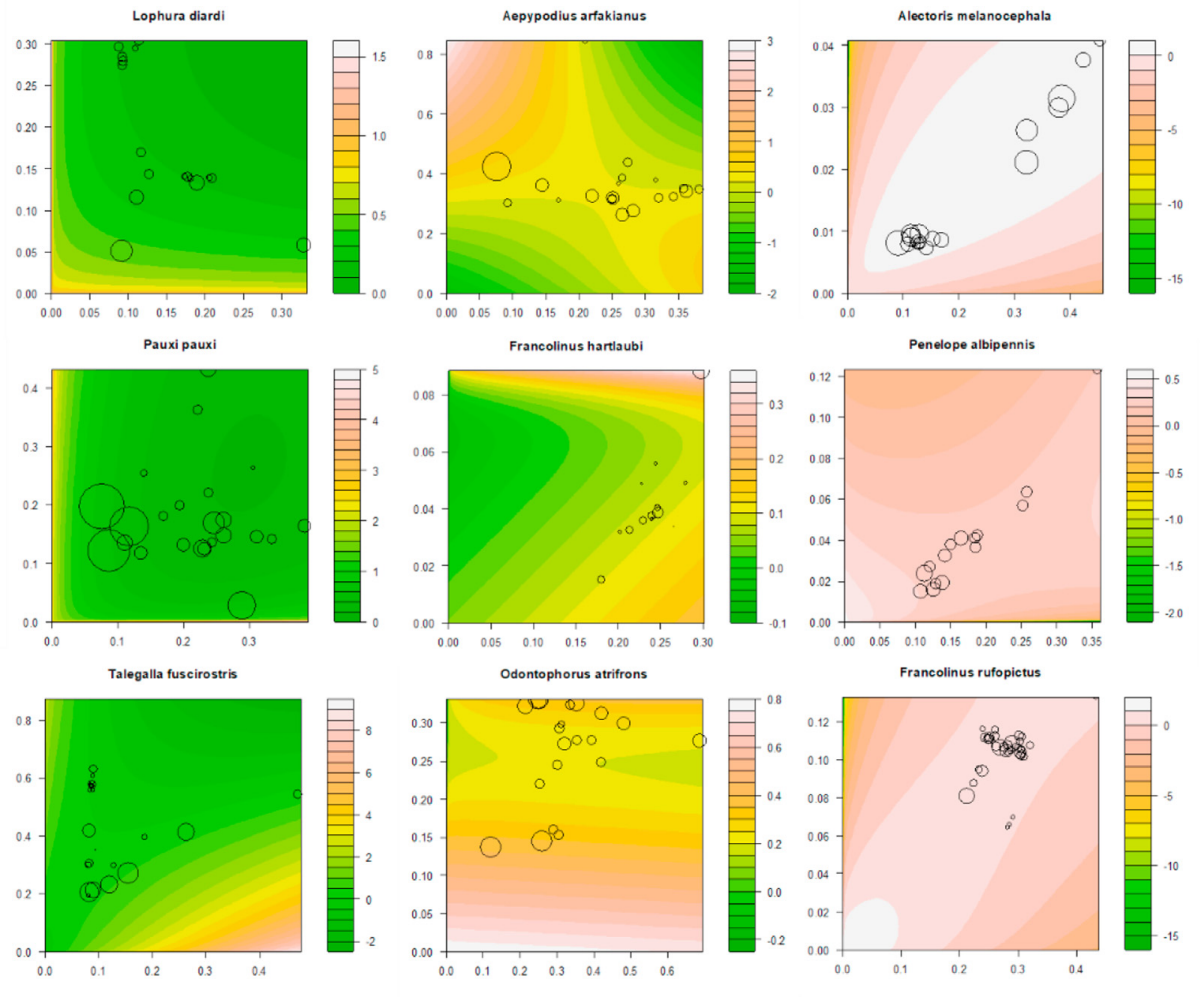
The observed human footprint index (represented by the sizes of the circle), the predicted human footprint index (represented by the colors) by FPIR, the values of elevation (x axis) and annual total precipitation (y axis) of the occurrences of nine Galliformes species with high interaction effects.

We selected nine Galliformes species, which had high interaction effects that contributed to over 25% of the total variance of the dependent variable (human footprint index), and showed that FPIRs were very flexible and fit various nonlinear patterns of the X-Y relationship well (Fig. 5).

### Why use FPIR?

For the nest site selection of the crested ibis, we found the birds selected the watersheds with higher areas of both rice paddies and waterbodies. The product of the areas of two wetland types (i.e. the interaction term) was the most important term in the species distribution model, and other variables such as elevation, precipitation, temperature, human impact, vegetation types all had trivial effect. We further developed FPIR and fit the model using the term X_1_^4.9^ X_2_^2.6^, and reached better performance than the regular regression.

Multiple linear regressions have been widely used for nearly a century [see 1,21], and they are primary methods for quantifying the relationship between a continuous dependent variable and several continuous explanatory variables [3]. The presence of interaction effects is common in regressions [4,^22^,23]. Back to 1923, Fisher and Mackenzie indicated potato yields were better fitted by a product formula than by a sum formula [8]. In 1936, Johnson & Neyman began to use the idea of "region of significance" to treat interaction [24]. Currently, the interaction effect, if it exists, is usually assumed to be of the form βX_1_X_2_, as defined in most text books [e.g. 2,3]. The potential difference of contribution to interaction effect between X_1_ and X_2_ has been ignored.

MFPI (multivariable fractional polynomials interaction) was introduced to quantify interactions using fractional polynomials, yet it focuses on multivariate analysis and only uses eight values to fit the exponents of the explanatory variables. In order to fit interaction in multiple regressions in a more powerful and parsimonious way, we developed the FPIR method to estimate exponent values (i.e. *M* and *N*) in the interaction term βX_1_^M^X_2_^N^. Our results indicated that FPIRs always performed better than regular multiple regressions. In fact, a regular multiple regression is one scenario of a FPIR when *M* = *N* = 1.

### What are the new results from FPIR?

FPIRs had lower AIC values than regular regression, and provide a more powerful way to quantify the interaction effect, using a series of exponential curves rather than straight lines for the X-Y relationship in the context of interaction (see Graphical abstract). Consequently, FPIRs helped identify hidden interaction effects that regular regression failed to detect.

FPIRs can provide more insights into real-world ecological questions. For the nest site selection of the crested ibis, the significant interaction effect identified that the bird relied on both rice paddies and waterbodies: if either rice paddies or waterbodies are not large enough in a watershed, the watershed typically remains unused for nesting. FPIRs further detected the unequal contribution of the two wetland types to the interaction and found that the rice paddies were more important than waterbodies in the nest site selection. Rice paddies had an optimal exponent of 4.9, about twice of that of waterbodies (2.6). The real situation was that the areas of rice paddies were about 10 times higher than that of waterbodies in 95 watersheds. Such results highlight the importance of rice paddies, which are the major foraging habitat during the breeding season, the most crucial stage in its life cycle. After the nestlings fledged, the birds moved to lower areas and foraged along waterbodies (i.e. rivers and ponds) [25]. From 1981 to 2013, the crested ibis population expanded from two breeding pairs [10] in two watersheds to 236 breeding pairs in 23 watersheds (Supplementary Fig. 1). During the early period of the recovery, the birds were found to have only stayed in the watersheds with high proportions of rice paddies [15], again supporting the key role of rice paddies in their population recovery.

### Advantages and weaknesses of FPIR

FPIRs produced lower *R^2^* values than complex models such as polynomial regressions, generalized additive models (GAMs), and random forest. For a regression with two explanatory variables, a fourth order polynomial regression has 15 terms, whereas a FPIR only has four terms. Polynomial regressions can quantify high order relationships between dependent and explanatory variables, whereas FPIRs behave like a local optimization (focusing on the interaction term rather than high-order terms) by ignoring the whole picture but fitting the interaction with higher accuracy. GAMs are more complex than polynomial regressions by using non-linear smooth functions to fit data [26], and they have numerous parameters and are hard understand . Random forest is even worse than GAMs in the aspect of transparency, as it uses many tree brunches to fit data and provides fragmented prediction surface (Fig. 2). Nevertheless, a FPIR is a linear model with a complex interaction term. It substantially improves the model fit over the regular multiple regression when the interaction term was significant. It is a parsimonious way to handle interacting continuous variables in regressions.

We used ranges of exponents *M* and *N* in FPIR Y ~ β_0_ + β_1X1_ + β_2X2_ + β_3_X_1_^M^ X_2^N^_ + ɛ from -56 to 56. The selection of those ranges was arbitrary. Compared with the default value of one generally assumed in regular multiple regressions, such ranges are much larger. We speculated such ranges would fit most situations. Nevertheless, the ranges of *M* and *N* can be easily expanded at the cost of more computation time.

The current version of FPIR can only quantify the interaction effect of continuous variables. It can not deal with the interaction involving categorical variables such as treatments, sites, groups, and so on. The package we provided for FPIR can process one two-way interaction, and one three-way interaction. Users would need to modify the code for other situations when more interaction terms exist.

Our new method enhances flexibility, interpretability and parsimony, while using a minimum number of degrees of freedom to maximize variances that are explained in the model. It has three advantages over the regular multiple linear regression: (1) it fits the model better with lower residuals than a regular regression; (2) it can evaluate the importance of two explanatory variables based on the values of their exponents in the interaction term; and (3) it can detect hidden interaction effects. While complex regression methods may explain more variations, they sacrifice the simplicity and readability, and tend to overfit the data [27]. Recognizing the potential for different explanatory variables to interact in nonlinear ways will help investigators to improve the identification of the different effects of explanatory variables on biological processes of interest. We suggest using FPIRs to fit models with different exponents for explanatory variables in interaction terms whenever the interaction terms are likely to be significant.

## Declaration of Competing Interest

The Authors confirm that there are no conflicts of interest.

## Appendix Supplementary materials

Image, application 1

Image, application 2

Image, application 3
