# Supplementary material for: Deeply digging the interaction effect in multiple linear regressions using a fractional-power interaction term
Source: MethodsX. 2020 Sep 16;7:101067. doi: 10.1016/j.mex.2020.101067 (PMC7549115; doi:10.1016/j.mex.2020.101067)
Supplement: Supplementary file 3 [file mmc3.docx]

**Supplementary Figures**

**
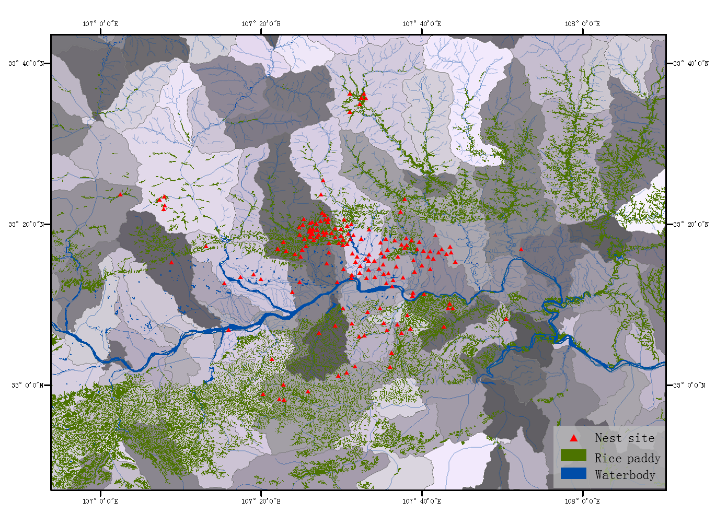
****Supplementary Fig. 1.** Distribution of the nests of crested ibis, rice paddies, and waterbodies in the watersheds in our study area (shadowed polygons) in Hanzhong, Shaanxi Province, China.

**Supplementary Fig. 2.** Distribution of 55 candidate values for the estimation of *M* and *N* in X_1_^M^ X_2_^N^ within FPIR.

**Supplementary Fig. 3.** Variance explained by the interaction terms in 4692 regular regressions and the corresponding FPIRs.
